# Universal scaling-law for flow resistance over canopies with complex morphology

**DOI:** 10.1038/s41598-018-22346-1

**Published:** 2018-03-13

**Authors:** Simonetta Rubol, Bowen Ling, Ilenia Battiato

**Affiliations:** Dept. of Energy Resources Engineering, Stanford University, Stanford, CA 94305 USA

## Abstract

Flow resistance caused by vegetation is a key parameter to properly assess flood management and river restoration. However, quantifying the friction factor or any of its alternative metrics, e.g. the drag coefficient, in canopies with complex geometry has proven elusive. We explore the effect of canopy morphology on vegetated channels flow structure and resistance by treating the canopy as a porous medium characterized by an effective permeability, a property that describes the ease with which water can flow through the canopy layer. We employ a two-domain model for flow over and within the canopy, which couples the log-law in the free layer to the Darcy-Brinkman equation in the vegetated layer. We validate the model analytical solutions for the average velocity profile within and above the canopy, the volumetric discharge and the friction factor against data collected across a wide range of canopy morphologies encountered in riverine systems. Results indicate agreement between model predictions and data for both simple and complex plant morphologies. For low submergence canopies, we find a universal scaling law that relates friction factor with canopy permeability and a rescaled bulk Reynolds number. This provides a valuable tool to assess habitats sustainability associated with hydro-dynamical conditions.

## Introduction

In the last decades, an increasing number of countries have introduced restoration practices to promote the sustainable management of rivers^1^. These practices often advocate the use of in-channel and riparian vegetation to preserve essential ecosystem services such as the reduction of banks erosion and the sheltering of fishes and invertebrates^2^. One key factor to properly assess water quality and ecological functions of rivers is the quantification of flow resistance induced by vegetation^3–5^. By creating an additional drag, canopies reduce the flow velocity in the vegetated layer, promote local retention of chemicals and deposition of particles that improve the stability of riverbanks and affect the fate and transport of nutrients and pollutants along the river^6–8^. While momentum and mass transfer mechanisms between the canopy and the free flows chiefly depend on vegetation geometry^9–14^, the characterization of friction in terms of vegetation morphology and flow regimes is an open challenge. Many studies have investigated the relationship of the drag coefficient *C*_*D*_ with Reynolds number^5,15–21^, stem population density^15,22,23^, canopy spacing^15,21^, plants geometrical arrangement^24,25^, blade flexibility^25,26^, leaf area to stem area ratio^26^, solid volume fraction^21,27,28^, penetration length^29–31^ and the Keuglan-Carpenter number for wave-dominated flows^16,32,33^. Yet, universal parametrizations of the drag force through a drag coefficient *C*_*D*_ have proven elusive due to its dependence on many parameters, notably the combination between canopy morphology and dynamic conditions. In alternative, empirical coefficients, e.g. Manning friction factor, can be used to estimate the flow resistance. However, their applicability is generally limited to the conditions in which they are originally estimated and their predictive capability has often been criticized, especially when dealing with complex channel and plant morphologies^34^. Natural vegetation can exhibit both relatively simple geometries (e.g. in grasses) and complex shapes as in plants with foliage, branches and leaves that create a vertical variability in the plant density^12^. This renders quantification of the compound effects of different plant components on flow resistance even more dire^20,35–37^. Leaves and branches increase the total surface area and the drag force^2,4,5^, while this effect decreases for flexible vegetation due to the reconfiguration of the plant in the streamline direction^38,39^. As a result, the relationship between the drag force and the mean flow velocity is in general linear or less than quadratic (with an exponent between 1.3 and 1.9)^4,19,40^.

A variety of flume studies with real plants or plant prototypes mimicking natural vegetation have been conducted to investigate the effects of natural vegetation on flow^38,39^. These studies suggest that the most important parameters affecting the flow resistance are the canopy density, the canopy porosity, the total surface area, the flexibility of the individual plants and community composition. Computational fluid dynamics (CFD) models, which explicitly resolve the flow field around individual stems, have increased our understanding of how different types of vegetation affect flow resistance and have allowed to quantify the impact that species-specific morphology has on fluid flow and friction.^37^. Yet, their upscaling from the stem- to the canopy-scale is not trivial due to the often prohibitive computational burden associated with modeling plant-plant interaction, such as the sheltering effect induced on one individual plant by the presence of others^37,41^. Instead, the majority of canopy-scale CFD models treat the canopy layer as an array of rigid cylinders, and their application to morphologically complex flexible canopies is questionable^42^. Only few sophisticated models (e.g., 3-D *κ* − *ε* turbulence models) account for canopy flexibility, but their computational cost prevents their use in design calculations^11,19^. Alternatively, simpler models and analytical solutions for flow over rigid canopies have the advantage to provide handy tools for engineers. However, as for the numerical models, their application to real flexible plants, rivers and estuaries has only been partially explored^42–44^. We refer to Nikora, *et al*.^45^ for a recent review of vegetated flow models.

Here, we employ the two-domain approach developed by Battiato and Rubol^11^ that couples the *log law* with the Darcy-Brikman equation for flow in the canopy layer to investigate (i) the impact of canopies with complex morphology on flow structure and flow resistance and, (ii) the capacity of a simple analytical solution to describe flow over and within flexible plants with complex structures. The model treats the canopy layer as a porous medium and, as a result, has a number of advantages: (i) the canopy layer morphology is uniquely characterized by one macroscopic parameter, i.e., the canopy effective permeability (a metric that quantifies how easily water flows through the vegetated layer), (ii) the model input parameters such as the height of the water level and the canopy can be directly estimated by remote sensing and acoustic techniques (i.e., echo sounder)^30,46^, and (iii) flow properties within and above the canopies (e.g. mean velocity field, friction factor and drag coefficient) are directly correlated to canopy morphology through its effective permeability and are also quantified by closed-form expressions. Treating canopy layers as porous media has already been successfully used to predict flow velocity and solute transport within and above obstructions^14,47–50^. First, we present the model and the closed-form expressions for the mean flow profile, the volumetric discharge and the friction factor. Then, we validate the model by comparing model predictions against experimental data collected across a wide range of rigid and flexible canopies commonly encountered in riverine systems, such as grasses, woody vegetation and bushes. Finally, we study how canopies with different shapes, flexibility and densities affect the flow resistance and uncover a universal *scaling*-*law* that describes the friction factor across different canopy morphologies.

## Model Description

**Figure 1:**
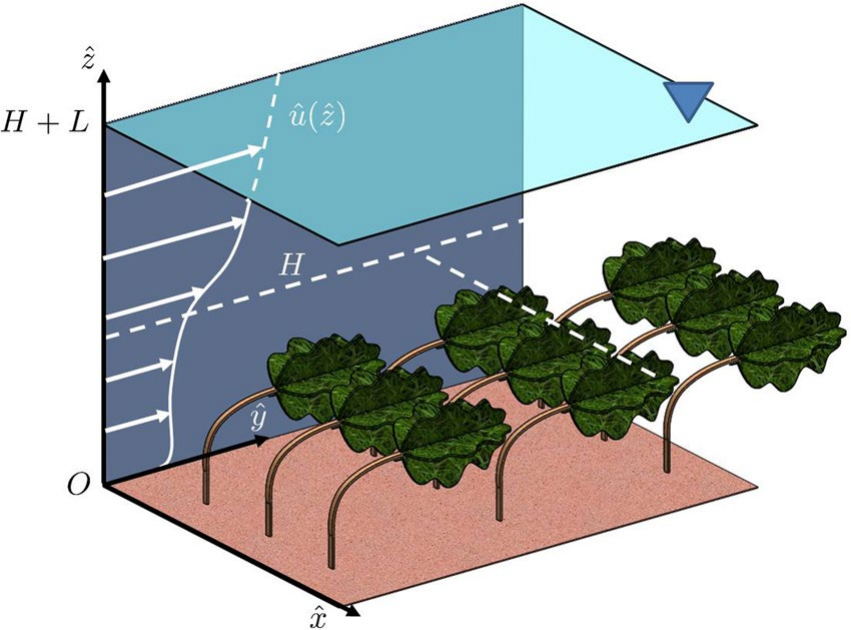
Sketch of the conceptual model. The deflected vegetated layer, of height *H*, is characterized by an effective permeability *K*, which describes the ease with which water can flow through the canopy layer. The water level above the canopy height is *L*, and *H* + *L* is the flow depth. The mean velocity profile above and within the canopy is $\hat{u}(\hat{z})$. The triangle indicates the location of the free surface, i.e. the air-water surface where the shear stress is zero.

Following the modeling framework described by Battiato and Rubol^11^, we consider a two-dimensional fully developed incompressible turbulent flow in an open channel of slope *θ* with *S*_0_ := tan*θ* ≈ sin*θ*, whose bottom part, $\hat{z}\in \mathrm{(0},\,H)$, is occupied by a morphologically complex canopy layer of (deflected) height *H*. The flow depth is *H* + *L*. A sketch of the model is shown in Fig. 1. The canopy is treated as a porous medium of permeability *K* [L^2^]. The effective permeability *K*, an intrinsic property of an obstruction and its morphology (i.e., porosity, density etc.), is a metric that describes how easily water flows through the canopy. The higher the permeability the lower is the resistance of the porous medium to flow. Similarly, the lower the permeability, the greater is the resistance to flow offered by the vegetation. In the context of classical porous media (e.g. consolidated and unconsolidated granular media like rocks and soils), there are numerous empirical relationships that allow one to estimate *K* from, e.g., porosity. One such famous relationship is the Karman-Cozeny equation. Yet, one should be cautious about the use of such empirical formulas to canopy layers since the structure of the obstruction is significantly different from that of geologic porous media (from spheroidal obstacles to fibrous-like obstacles)^51^. We emphasize that the link between canopy permeability *K* and geometrical features of the vegetated layer (e.g. canopy density, porosity, leaf area density etc.) has been explored by Battiato and Rubol^11^ for canopies constituted by regular arrays of rigid cylinders. The extension to flexible canopies with complex morphology is object of current investigations. For completeness, we report the model equations developed by Battiato and Rubol^11^. The mean velocity profile above and within the canopy layer is obtained by coupling the *log*-*law*

1 $$\hat{u}(\hat{z})=\hat{U}+\frac{{\hat{u}}_{\tau }}{\kappa }\,{\rm{l}}{\rm{n}}(\frac{\hat{z}}{H}),\quad \hat{z}\in (H,H+L),$$

to the Darcy-Brinkman equation

2 $${\mu }_{e}\frac{\,d{}^{2}\hat{u}(\hat{z})}{\,d\,{\hat{z}}^{2}}-\frac{{\mu }_{e}}{K}\hat{u}(\hat{z})+\rho g{S}_{0}=\mathrm{0,}\quad \hat{z}\in \mathrm{(0},H),$$

with no shear condition at the channel bottom, i.e. $\hat{\tau }\mathrm{(0)}=0$, and continuity of velocity and shear stress at the interface between the free and canopy flows, i.e. $\hat{u}({H}^{-})=\hat{u}({H}^{+})=\hat{U}$ and ${\mu }_{e}\,\,{d}_{\hat{z}}\hat{u}{|}_{{H}^{-}}={\mu }_{t}({H}^{+}){d}_{\hat{z}}\hat{u}{|}_{{H}^{+}}$, where $\hat{U}$ is the (mean) velocity at the top of the canopies, ${\hat{u}}_{\tau }=\sqrt{g{S}_{0}L}$ is the friction velocity, *μ*_*e*_ is the fluid “effective” viscosity, set equal to the turbulent viscosity, i.e. ${\mu }_{e}\,:={\mu }_{t}=\rho \kappa H{\hat{u}}_{\tau }$, and *κ* is the reduced von Kármán constant^52,53^ (see^11^ for details). We define the following dimensionless quantities

3 $$z=\frac{\hat{z}}{H},\quad u=\frac{\hat{u}}{q},\quad \delta =\frac{L}{H},\quad U=\frac{\hat{U}}{q},\quad {\lambda }^{2}=\frac{{H}^{2}}{K},$$

where *q* = *ρgS*_0_*H*^2^/*μ*_*e*_ is a characteristic velocity scale. The analytical solution of Eqs (1–2) for the mean velocity profile $\hat{u}(z)$ inside and above the canopy layer is^11^

4a $$\hat{u}(z)=q[{\lambda }^{-2}+C({e}^{\lambda z}+{e}^{-\lambda z})],\quad z\in \mathrm{(0,}\,\mathrm{1],}$$

4b $$\hat{u}(z)=q[U+\delta \,\mathrm{ln}\,z],\quad z\in \mathrm{(1},1+\delta ),$$

respectively, where

5a $$C=\frac{1}{2}\delta {\lambda }^{-1}\,\mathrm{csch}\,\lambda ,$$

5b $$U={\lambda }^{-2}+\delta {\lambda }^{-1}\,\coth \,\lambda ,$$

and $\hat{U}\,:=qu\mathrm{(1)}$ is the interfacial velocity. The volumetric discharge $\hat{Q}$ [L^3^T^−1^] through a vegetated channel of width *B* [L] can be determined from direct integration of Eq. (4),

6 $$\hat{Q}=qHB\{{\lambda }^{-2}+C{\lambda }^{-1}({e}^{\lambda }-{e}^{-\lambda })+\delta [(1+\delta )ln(1+\delta )+U-\delta ]\mathrm{\}}.$$

The Darcy friction factor *f* is defined as

7 $$f\,:=8\frac{\hat{\tau }(H)}{\rho {\hat{U}}_{b}^{2}}$$

where $\hat{\tau }(H)=\rho g{S}_{0}L$ and ${\hat{U}}_{b}$ is the bulk velocity defined as

8 $${\hat{U}}_{b}\,:=\frac{\hat{Q}}{(H+L)B}.$$

Among the relevant parameters that are used to quantify the flow resistance, we report the expression for the inverse of the drag length scale *C*_*D*_*a*, given by the product of the medium drag coefficient, *C*_*D*_, and the frontal area of the canopies, *a*

9 $${C}_{D}a\,:=\frac{1}{H}\frac{2\delta }{U}{(\lambda \kappa )}^{2}.$$

Once the channel morphology (i.e. *H*, *L*, *B*, *S*_0_) and the canopy effective properties (i.e. its permeability *K*) are known, Eqs (4), (6) and (7) can be used to analytically calculate/predict the mean velocity profile above and within the canopy $\hat{u}(z)$, the volumetric discharge $\hat{Q}$ and the Darcy friction factor *f*.

In the following Section, we compare model predictions and experimental data published in the literature.

## Results and Discussion

In this Section, first we present a comparison between model predictions and experimental measurements collected from the literature, and then identify a universal scaling law for the friction factor across different canopy morphologies. The data include velocity profiles, flow rates and friction factors collected both in flumes and real channels, and span a variety of vegetation types and morphologies ranging from grasses (e.g., eelgrass, cordgrass and barley) to woody vegetation (e.g., poplars) and bushes (e.g., willows), with plant heights varying from 0.05 m to 0.7 m, channel slopes between 0.03–23 % and submergence (defined as the ratio between the flow depth and the canopy height, i.e. (*H* + *L*)/*H*) ranging from 1.25 to 23. The dataset names, and their corresponding references, are listed in Table 1, together with relevant parameters of the experimental setups (i.e., *H*, *L*, *S*_0_ and *B*). A more detailed description of the type of vegetation included in the dataset can be found in the Methods Section.

**Table 1:** Parameters of the experimental datasets considered in this study with the corresponding reference.

Authors	Dataset	*H*[m]	*L*[m]	*S*_0_[−]	*B* [m]	(*H* + *L*)/*H*[−]
Nepf and Vivoni^55^	NV125	0.16	0.04	2.80E-04	0.38	1.25
	NV150	0.16	0.08	2.50E-04	0.38	1.50
	NV175	0.16	0.12	2.50E-04	0.38	1.75
	NV190	0.16	0.14	5.00E-04	0.38	1.90
	NV275	0.16	0.28	2.00E-04	0.38	2.75
Wilson, *et al*.^63^	WF	0.11	0.1	1.00E-03	0.5	1.95
	WF2	0.11	0.18	1.00E-03	0.5	2.75
Velasco, *et al*.^59^	VELASCO	0.14	0.12	1.35E-01	2.5	1.84
	VELASCO2	0.13	0.09	1.10E-01	2.5	1.74
Baptist^62^	BR2	0.09	0.22	1.37E-01	0.8	3.40
	BR3	0.09	0.2	1.01E-01	0.8	3.19
Righetti^57^	RSparse	0.45	0.48	1.53E+00	2	2.07
	RDense	0.58	0.32	1.31E+00	2	1.55
Shucksmith, *et al*.^61^	Shuck7	0.16	0.07	2.80E-02	0.6	1.46
	Shuck10	0.18	0.08	2.80E-02	0.6	1.43
Shi, *et al*.^60^	Shi20	0.06	0.27	3.30E-03	0.3	5.53
	Shi40	0.12	0.22	3.90E-03	0.3	2.85
	Shi60	0.18	0.16	2.80E-03	0.3	1.88
Siniscalchi *et al*.^58^	SiniscalchiH	0.23	0.06	1.20E-01	0.6	1.26
	SiniscalchiM	0.23	0.06	8.10E-02	0.6	1.26
Cassan, *et al*.^56^	CassanS11	0.08	1.6	4.00E-04	1.3	21.0
	CassanS12	0.05	1.1	4.00E-04	1.3	23.0
	CassanS21	0.09	1.13	1.30E-03	0.8	13.6
	CassanS22	0.08	1.17	1.30E-03	0.8	16.6

The parameters *H*, *L*, *S*_0_ and *B* are the height of the canopies, the depth of the free-surface layer, the channel slope and the width of the channel, respectively. The parameter (*H* + *L*)/*L* is the canopy submergence.

### Velocity profiles

To test the capability of the proposed model to reproduce the flow within and above canopies with different morphologies and flexibilities, we first compare the analytical solution in Eq. (4) to the experimental datasets listed in Table 1.

**Figure 2:**
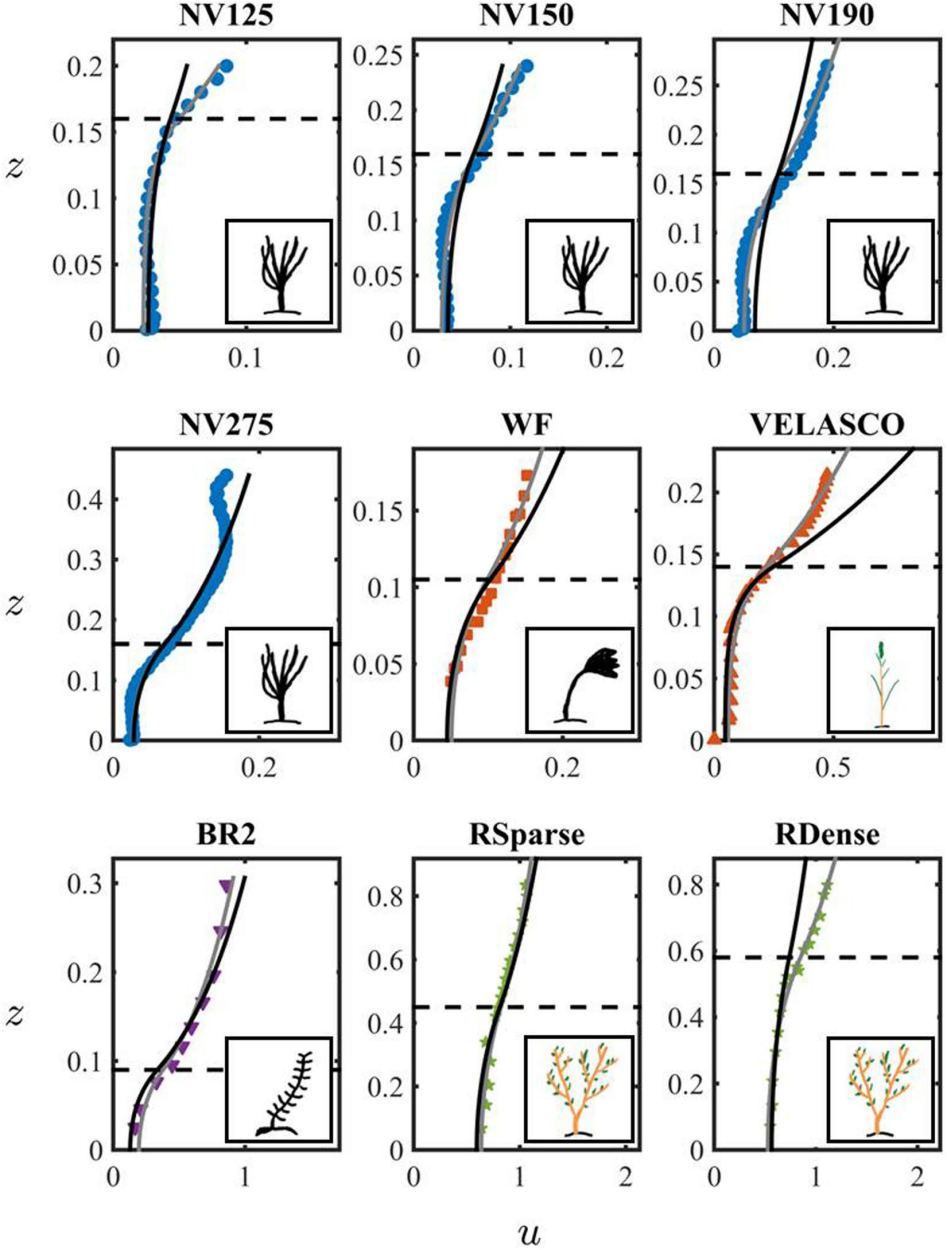
Comparison between measured (symbols) and modeled (lines) velocity profiles for the datasets by Nepf & Vivoni^55^ (NV125, NV150, NV190 and NV275), Wilson *et al*.^63^ (WF), Velasco *et al*.^59^ (VELASCO), Baptist^62^ (BR2) and Righetti^57^ (RSparse and RDense) of Table 1. In the Figure, *u* is the dimensionless mean velocity, *z* is the dimensionless coordinate. Their definition is provided in Eq. (3). The solid black and grey lines correspond to the dimensionless mean velocity profile *u* predicted by (4) using one and and two fitting parameters, respectively. The list of parameters used for the fitting is provided in Table 2. The insets contain sketches of the canopy used in each experimental study: color and black drawings indicate real and plastic vegetation, respectively.

**Figure 3:**
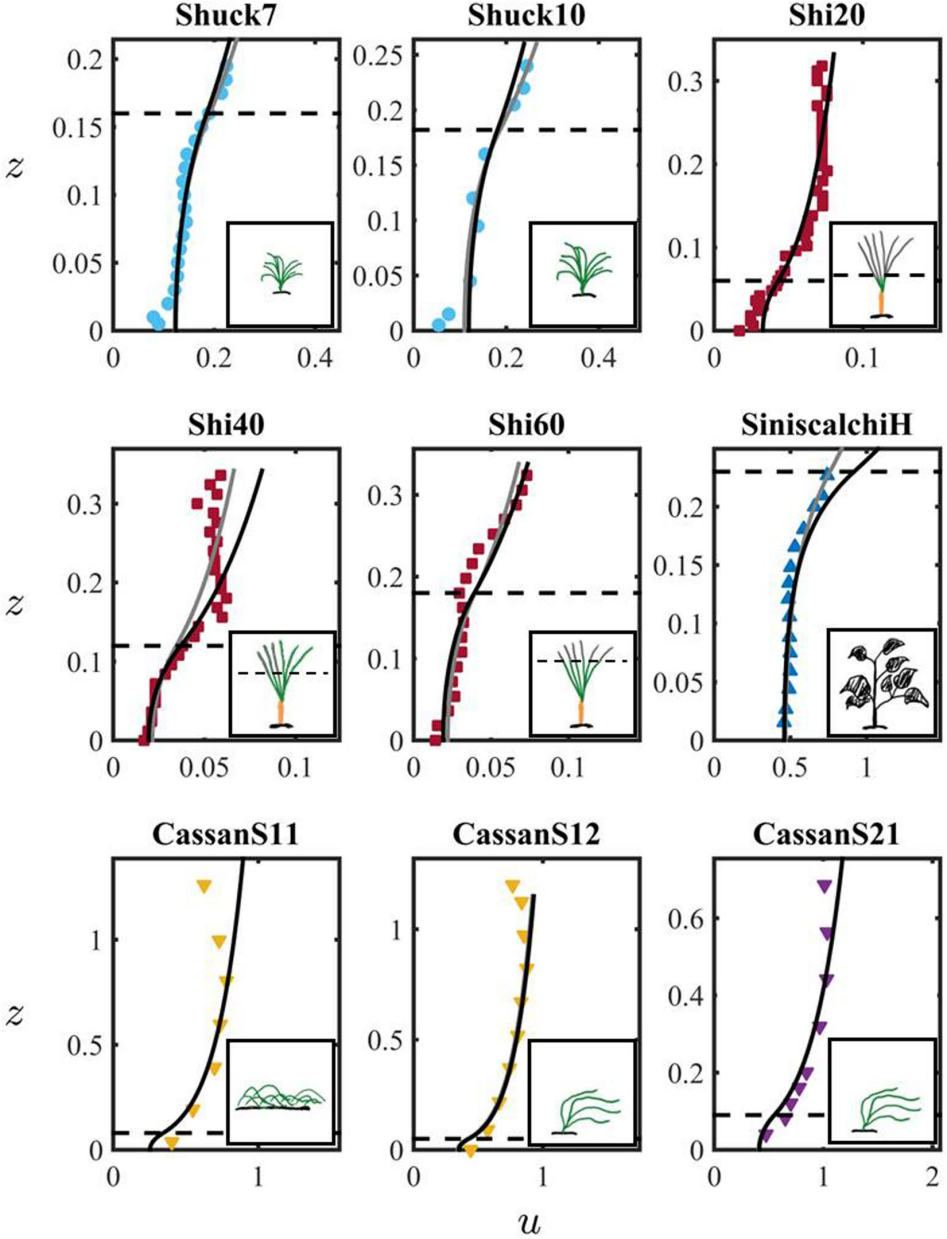
Comparison between measured (symbols) and modeled (lines) velocity profiles for the datasets by Shucksmith *et al*.^61^ (Shuck7 and Shuck10), Shi *et al*.^60^ (Shi20, Shi40 and Shi60), Siniscalchi *et al*.^58^ (SiniscalchiH), and Cassan *et al*.^56^ (CassanS11, CassanS12 and CassanS21) of Table 1. In the Figure, *u* is the dimensionless mean velocity, *z* is the dimensionless coordinate. Their definition is provided in Eq. (3). The solid black and grey lines correspond to the dimensionless mean velocity profile *u* predicted by (4) using one and and two fitting parameters, respectively. The list of parameters used for the fitting is provided in Table 2. The insets contain sketches of the canopy used in each experimental study: color and black drawings indicate real and plastic vegetation, respectively.

Figures 2 and 3 show the comparison between the measured and predicted velocity profiles. The comparison is conducted by fitting either one (i.e. canopy permeability, *K*) or two (i.e. canopy permeability, *K*, and the reduced von Kármán constant, *κ*) parameters using the orthogonal distance algorithm (ODA^54^), which minimizes the orthogonal distance between the model curve and the experimental data (solid black and grey lines for the one and two parameter fitting, respectively). Figures 2 and 3 show that the model (both with 1 and 2 fitting parameters) successfully reproduces the mean velocity profiles in the canopy layer across a wide range of canopy morphologies for both real plants and plastic prototypes. Additionally, surface layer velocity predictions with the one-fitting parameter model improve with increasing submergence, as shown in the profiles of Nepf and Vivoni^55^ (NV125, NV150, NV175, NV190 and NV275) in Fig. 2 and Cassan, *et al*.^56^ (CassanS11, CassanS12, CassanS21, CassanS22) in Fig. 3. Remarkably, despite the model is developed under the hypothesis of non-deformable media, it is still capable to well reproduce the velocity profiles measured in flexible vegetation, including tall willows, i.e. RSparse and RDense in Fig. 2 (dataset of Righetti, *et al*.^57^), in patches of poplars, i.e. SiniscalchiH in Fig. 3 (dataset of Siniscalchi, *et al*.^58^), as well as the velocity profiles collected by Cassan, *et al*.^56^ in a real channel with prone non-uniform vegetation, i.e. CassanS11, CassanS12, CassanS21, CassanS22 in Fig. 3.

While the one-fitting parameter model always successfully captures the flow field in the canopy layer for the whole dataset of Figs 2 and 3, the two-fitting parameter model is necessary to improve the description of the velocity field in free-surface layer in channels where the water height above the canopy constrains the development of the *log*-*law* (see the profiles RDense of Righetti^57^, NV125 of Nepf, *et al*.^55^ and VELASCO of Velasco^59^ in Fig. 2). This is expected since for low submergence the boundary effects at the surface layer become important throughout the flow domain, and an additional fitting parameter (*κ*) is needed to compensate for the lack of accuracy of the *log law* at the free surface. The average value of the von Kármán constant in the two-fitting parameter model is *κ* = 0.22 (for the low submergence cases), only slightly higher than the value *κ* = 0.19 used for the one-fitting parameter model. While an open debate still exists on the universality of the von Kármán constant, we find that the fitted values are consistent with the values measured in vegetated flows^53^ (see^11^ for additional discussion). Instead, one fitting parameter is sufficient when the water height above the canopy is larger than the canopy height (i.e., $\delta :=L/H > 1$). Note that the velocity profiles of Cassan, *et al*.^56^ (i.e. CassanS11, CassanS12, CassanS21, and CassanS22) refer to highly submerged canopies (i.e., submergence greater than ten) for which the velocity profile is characterized by boundary layer rather than mixing layer features^2^. The model well reproduces the shape of the measured profiles with only slight deviations for points in proximity of the free surface, for which presence of secondary currents causes a maximum of velocity below the air-water interface (see Cassan *et al*.^56^ for details). Similarly, deviations between the data and the two-fitting parameter model were observed close to the free surface for the Shi40 dataset^60^ in Fig. 3 and the NV275 dataset^55^ in Fig. 2, where the experimental profiles capture the no-stress condition on the free surface^2^. We emphasize that these deviations between data and model are expected since the *log law* is not accurate at the air-water interface. While the model is able to capture the velocity profiles of flexible vegetation considered in this study, it cannot, in its current form, predict secondary maxima of velocity due to strong vertical variability in plant density induced, e.g., by reconfiguration. Nevertheless, the model can be generalized to account for vertical heterogeneity and poro-elasticity by (i) assuming that permeability varies along the vertical direction (e.g., *K* = *K*(*z*)) and (ii) coupling the plant deflection with the flow field, following an approach similar to that proposed by Battiato, *et al*.^47^, who modelled the bending of carbon nanotubes forests under aerodynamic shear.

The fitted values of permeability *K* vary in the range 10^−4^–10^−1^ m^2^ or between 0.6–5 in term of the inverse dimensionless permeability, $\lambda \,:=H/\sqrt{K}$. As expected, low permeable porous media are associated to highly dense canopy as for the dataset VELASCO^59^ in Fig. 2, while highly permeable media correspond to the sparse willows of Righetti^57^ (RSparse and RDense in Fig. 2). Shucksmith, *et al*.^61^ (datasets Shuck7 and Shuck10 of Fig. 3) measured the velocity profiles at different stages of the plant growth and found that the velocity inside the channel decreased as the plant height and the plant density increased. This is also consistent with the increase in friction values described in the following section. A similar effect was also found for the data of Shi^60^ (Shi20, Shi40 and Shi60 in Fig. 3). Changes in permeability due to changes in plant density were observed also for the dataset RSparse and RDense of Righetti^57^, for which the permeability values more than halved from the dense (2.5 bushes per square meter for the RDense dataset) to the sparse (1.1 bushes per square meter for the Rsparse dataset) configuration of willows. A summary of the relevant data including the value of the fitting parameters and the error between the analytical model and the experiments is provided in Table 2.

**Table 2:** List of fitting parameters in Eq. (4) for each dataset of Table 1.

One Fitting Parameter						Two Fitting Parameters				
Data	*κ*[−]	*K*[m^2^]	*λ*[−]	Λ[−]	Error	*κ*[−]	*K*[m^2^]	*λ*[−]	Λ[−]	Error
NV125^55^	0.19	2.80E-03	3.00	0.75	1.29E-01	7.87E-02	1.07E-03	4.89	1.22	8.86E-02
NV150^55^	0.19	4.98E-03	2.27	1.13	1.60E-01	1.23E-01	2.85E-03	3.00	1.50	8.73E-02
NV175^55^	0.19	4.98E-03	2.27	1.70	1.25E-01	**1**.**23E-01**	**2**.**85E-03**	**3**.**00**	**2**.**25**	**6**.**63E-02**
NV190^55^	0.19	1.05E-02	1.56	1.41	2.66E-01	1.64E-01	4.98E-03	2.27	2.04	8.12E-02
NV275^55^	0.19	5.80E-03	2.10	3.68	1.21E-01	1.88E-01	5.61E-03	2.14	3.74	1.14E-01
WF^63^	0.19	2.00E-03	2.35	2.24	1.01E-01	2.51E-01	2.80E-03	1.98	1.89	6.75E-02
WF2^63^	0.19	6.43E-04	4.14	7.36	1.97E-01	**2**.**51E-01**	**2**.**80E-03**	**1**.**98**	**3**.**48**	**1**.**54E-02**
VELASCO^59^	0.19	6.43E-04	5.52	4.61	1.40E-00	3.26E-01	1.29E-03	3.90	3.26	1.61E-01
VELASCO2^59^	0.19	5.94E-04	5.21	3.86	1.76E-01	**3**.**26E-01**	**1**.**29E-03**	**3**.**53**	**2**.**62**	**1**.**48E-01**
BR2^62^	0.19	2.00E-03	2.01	4.84	1.12E-01	2.36E-01	3.30E-03	1.57	3.76	8.08E-02
BR3^62^	0.19	1.63E-03	2.23	4.88	1.43E-01	**2**.**36E-01**	**3**.**30E-03**	**1**.**57**	**3**.**43**	**5**.**62E-02**
RSparse^57^	0.19	1.43E-01	1.19	1.27	4.14E-02	2.28E-01	1.80E-01	1.06	1.13	2.58E-02
RDense^57^	0.19	1.87E-01	1.34	0.74	9.19E-02	9.08E-02	9.01E-02	1.93	1.07	3.03E-02
Shuck7^61^	0.19	7.31E-03	1.87	0.86	8.74E-02	1.64E-01	6.31E-03	2.01	0.93	8.43E-02
Shuck10^61^	0.19	8.46E-03	1.98	0.85	2.51E-01	1.39E-01	5.93E-03	2.36	1.01	2.04E-01
Shi20^60^	0.41	4.88E-03	0.86	3.89	8.91E-02	4.06E-01	4.81E-03	0.87	3.92	8.84E-02
Shi40^60^	0.19	5.45E-03	1.63	3.01	1.57E-01	2.69E-01	7.94E-03	1.35	2.49	1.13E-01
Shi60^60^	0.19	6.86E-03	2.17	1.91	1.49E-01	2.32E-01	9.20E-03	1.88	1.65	1.30E-01
SiniscalchiH^58^	0.19	3.16E-03	4.09	1.07	6.91E-02	3.90E-01	6.31E-03	2.90	0.76	3.79E-02
SiniscalchiM^58^	0.19	3.76E-03	3.75	0.98	9.43E-02	**3**.**90E-01**	**6**.**31E-03**	**2**.**90**	**0**.**76**	**5**.**83E-02**
CassanS11^56^	0.41	8.91E-03	0.85	17.0	1.49E-01	4.10E-01	8.91E-03	0.85	17.0	1.49E-01
CassanS12^56^	0.41	5.61E-03	0.67	14.7	2.95E-02	4.10E-01	5.46E-03	0.68	14.9	3.14E-02
CassanS21^56^	0.41	1.18E-03	0.83	10.4	8.61E-02	4.06E-01	1.15E-02	0.84	10.5	9.02E-02
CassanS22^56^	0.41	8.41E-03	0.82	12.8	1.11E-01	**4**.**06E-01**	**8**.**70E-03**	**0**.**70**	**10**.**9**	**1**.**07E-01**

**Figure 4:**
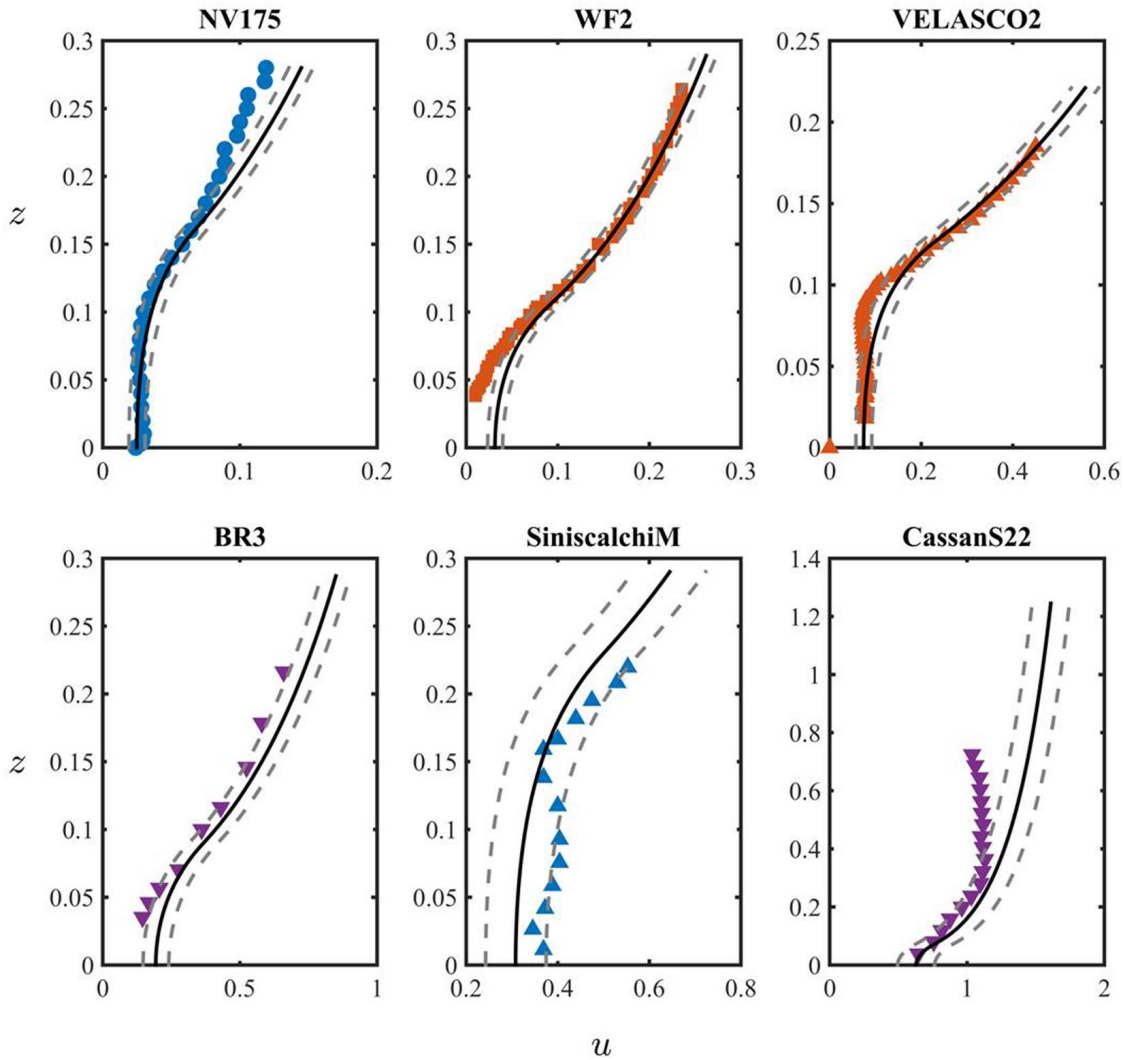
Comparison between measured (symbols) and fit-free modeled (lines) velocity profiles for the datasets by Nepf & Vivoni^55^ (NV175), Wilson *et al*.^63^ (WF2), Velasco^59^ (VELASCO2), Baptist^62^ (BR3), Siniscalchi *et al*.^58^ (SiniscalchiM) and Cassan *et al*.^56^ (CassanS22) of Table 1. In the Figure, *u* is the dimensionless mean velocity, *z* is the dimentionless coordinate. Their definition is provided in Eq. (3). The solid black line corresponds to the dimensionless mean velocity profile *u* predicted by (4) without fitting parameters. The dashed grey lines represent the model predictions with a ±10% uncertainty in the permeability estimate. The parameters used for fit-free predictions are provided in bold in Table 2.

The left portion of Table 2 provides the parameters values resulting from fitting the canopy permeability *K* only. The right portion of the Table 2 lists the parameters values resulting from fitting both the canopy permeability *K* and the reduced von Kármán constant *κ*. The parameters *λ* and Λ are defined as $\lambda \,:=H/\sqrt{K}$ and ${\rm{\Lambda }}\,:=L/\sqrt{K}$. The error between the analytical model (4) and the experimental mean velocity data is also provided. The numbers in boldface refer to the parameters values used for fit-free predictions of the mean velocity profiles of Fig. 4. The visual comparison between model predictions and experimental data is provided in Figs 2 and 3 for the fitted velocity profiles, and in Fig. 4 for the fit-free predictions.

To further test the predictive capabilities of Eq. (4), we use the model calibrated on six datasets from Figs 2 and 3 (NV150, WF, VELASCO, BR2, SiniscalchiH and CassanS21) to perform a series of fit-free predictions on a different set of data (NV175, WF2, VELASCO2, BR3 and SiniscalchiM, CassanS22, respectively), i.e. we use calibrated permeability and von Kármán constant values to perform fit-free predictions of the mean velocity profiles collected for plants with the same morphology but under different dynamical conditions (i.e. water level and volumetric discharge). Figure 4 illustrates the comparison between the measured and the predicted mean velocity profiles, and shows good agreement within the uncertainty level (10% of the permeability value) for both morphologically simple (e.g., NV175, BR3) and complex (e.g., WF2, SiniscalchiM) canopies. Predictions of the velocity profiles within canopies with simple morphologies (e.g., BR3^62^) were more accurate than those for morphologically complex canopies (e.g., SiniscalchiM^58^ and WF2^63^). This is expected because the projected area (and therefore, the permeability) of plants with complex morphology is more sensitive to changes in water level than plants with simpler shape. This is due to the presence of leaves and branches that become more streamlined as velocity increases^64–66^.

### Discharge and Friction Factor

**Figure 5:**
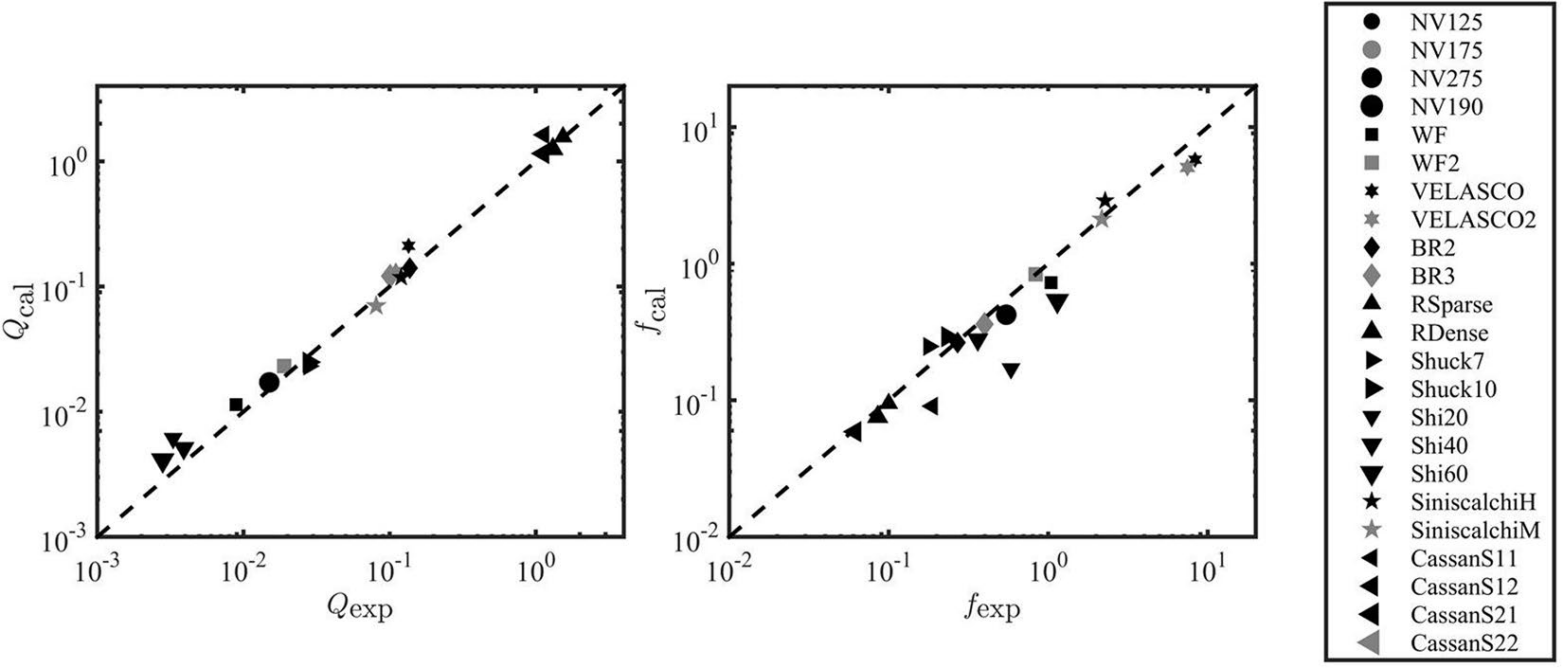
(Left) Comparison between the experimentally measured discharge *Q*_*exp*_ and the one calculated through Eq. (6), *Q*_*cal*_, for the datasets of Table 1. The black symbols corresponds to the one-fitting parameter predictions, while the grey symbols corresponds to *Q*_*cal*_ estimates without fitting parameters. The dashed black line is the 1:1 line. (Right) Comparison between the experimentally measured friction factor *f*_*exp*_ and the one calculated through Eq. (7), *f*_*cal*_, for the datasets of Table 1. The black symbols corresponds to the one-fitting parameter predictions, while the grey symbols corresponds to *f*_*cal*_ estimates without fitting parameters. The dashed black line is the 1:1 line.

Once the fitted (Figs 2 and 3) and predicted (Fig. 4) velocity profiles are determined, we use Eq. (6) to calculate the discharge. Figure 5(left) shows the comparison between the measured and predicted discharge, *Q*_*exp*_ and *Q*_*cal*_, respectively. The grey symbols refer to the purely predicted discharge (i.e. no fitting parameters) obtained from the profiles of Fig. 4, while the remaining symbols have been obtained with the one-fitting parameter model (i.e. solid black lines in Figs 2 and 3). The good agreement between data and predictions demonstrates that the one-fitting parameter model is sufficient to predict the flow rate in vegetated channels with sufficient accuracy. The two-parameter model can be used when highly precise measurements of the flow profile are required.

The vegetative resistance, i.e. the Darcy friction factor *f* (=*C*_*f*_/4), can be estimated from Eq. (7). Figure 5(right) shows a good agreement between the experimental and analytically predicted Darcy friction factors, *f*_*exp*_ and *f*_*cal*_, respectively. We emphasize that, in this formulation, the information about the canopy morphological complexity is entirely accounted for through the effective canopy permeability *K* (or its dimensionless counterpart *λ*^−1^). As a result, *f* depends on canopy morphology through *K* and its direct effect on modulating the flow rate $\hat{Q}$ in the channel as evident from Eq. (6). Maximal values of friction factor are obtained for the plastic prototypes and for the highly dense (22500 stem/m^2^) canopy of Velasco *et al*.^59^ (VELASCO and VELASCO2). Among the prototypes, the canopies with more complex morphology, as the poplars of Siniscalchi^58^ (SiniscalchiH and SiniscalchiM) and the seegrasses of Wilson^63^ (WF and WF2), result in a greater resistance to the flow than the ones with simpler geometry as the meadows of Nepf & Vivoni^55^ (NV125, NV175, NV190 and NV275). The lower friction factor for the willows of Righetti^57^ (RSparse, RDense) is explained by the higher flexibility and the smaller dimension of the leaves comparing to Siniscalchi^58^’s canopies (SiniscalchiH and SiniscalchiM). Minimal values of *f* were obtained for the deflected plants of Cassan^56^ (CassanS11, CassanS12, CassanS21, CassanS22). This is in line with the experimental work of Velasco^67^, that reported minimum resistance to the flow for highly deflected vegetation, with a friction factor comparable to the value of non-vegetated channel, *f* = 0.15, measured in gravel beds. The above considerations are also consistent with the medium drag *C*_*D*_*a* values calculated from (9). Noticeably, the computed values fall within the same order of magnitude of the values reported by Vargas^42^(see their Table 4), with higher medium drag for grasses than shrubs and forests. Also, the increase of shear stress with increasing plant height observed in the dataset of Shi^60^ (Shi20, Shi40 and Shi60) is in agreement with the increase of the Darcy friction factors.

### Universal Scaling of the Friction Factor

A number of works have pointed out that dynamic similarity can be often observed in systems characterized by coupled flows through and over permeable media^11,48,68,69^. After model validation, we use the porous medium analogy to investigate the existence of such a universal scaling law for vegetated flows across different canopies morphologies. Our main result is the derivation, detailed in the Methods section, of the universal scaling between the Darcy friction factor, Reynolds number and canopy permeability.

**Figure 6:**
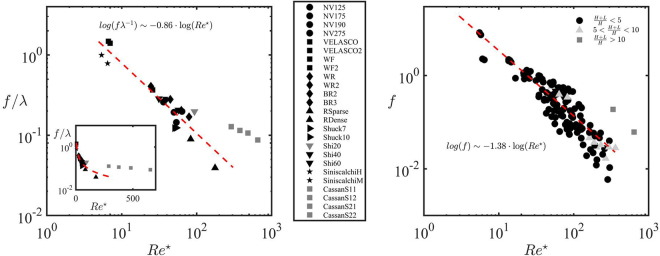
(Left) Validation of the universal scaling law (13) for thick canopies, i.e. canopies with low submergence. Experimental (symbols) and predicted (dashed line) universal scaling between *f*/*λ* and $R{e}^{\star }$ given in Eq. (13) for the dataset of Table 1: *f* is the Darcy friction factor, defined in Eq. (7), $\lambda \,:=H/\sqrt{K}$ is the dimensionless inverse canopy permeability and $R{e}^{\star }$ is the rescaled Reynolds number defined in Eq. (14). The black and grey symbols correspond to thick (low submergence) and thin (high submergence) canopies, respectively. The inset shows the same comparison is a semilog scale. The analytical interpolant is ${\rm{l}}{\rm{o}}{\rm{g}}(f/\lambda )=-0.86\,{\rm{l}}{\rm{o}}{\rm{g}}\,R{e}^{\star }+1.74$. (Right) Validation of the simplified universal scaling law (15) for thick canopies, i.e. canopies with low submergence. Experimental (symbols) and predicted (dashed line) universal scaling for a larger dataset including experiments from Table 1 of Poggi *et al*.^30^ and the ones collected in the Babolrod river by Miyab *et al*.^70^ in addition to the data reported in Table 1. The dashed line corresponds to the interporlant of equation ${\rm{l}}{\rm{o}}{\rm{g}}(f)=-1.38\,{\rm{l}}{\rm{o}}{\rm{g}}\,R{e}^{\star }$. Black, light grey and dark points indicate canopies with low, intermediate and high submergence, respectively.

The drag coefficient is defined as

10 $${C}_{D}\,:=\frac{2D}{\rho {\hat{U}}_{b}^{2}{ {\mathcal L} }^{2}},$$

where *D* is the drag force and ${\mathcal L}$ is a characteristic length scale of the system. Using Bunckingham’s Pi theorem and postulating the existence of self-similarity of the second kind (see Methods Section), we find that *C*_*D*_ scales a

11 $${C}_{D}\sim \frac{{\lambda }^{\alpha }}{R{e}^{\star }},$$

when the canopy layer is thick (or for shallow submergence), i.e. ${\rm{\Lambda }}:=\delta \lambda =L/\sqrt{K}\le 1$ (see Methods Section for detailed derivation). In (11),

12 $$\lambda \,:=\frac{H}{\sqrt{K}},\quad \quad R{e}^{\star }=\frac{\rho {\hat{U}}_{b}{\mathscr{L}}}{{\mu }_{t}},\quad \quad {\mu }_{t}=\rho \kappa H\sqrt{g{S}_{0}L},$$

and $\alpha  > 0$ is an unknown exponent. The former scaling suggests that (i) the drag force scales linearly with the mean flow velocity and the turbulent viscosity and (ii) the friction factor increases with decreasing canopy permeability (i.e., with increasing values of *λ*) since denser canopies are less permeable and offer greater blockage to the flow. To test the proposed scaling (11) under the hypothesis that most of the resistance offered by vegetation is due to friction (i.e. ${C}_{D}\sim f$), we plot *f*/*λ* as a function of $R{e}^{\star }$ where *f* is the experimentally measured friction factor, *α* = 1 and ${\mathcal L} =H+L$, see Fig. 6(left). While it is expected that the global resistance in vegetated channels decreases with the $R{e}^{\star }$ number, as plants become more streamlined to the flow, Fig. 6(left) shows that the friction factor exhibits the universal scaling proposed in (11) across different canopy morphology. Specifically, *f*/*λ* scales with the inverse of $R{e}^{\star }$, the modified Reynolds number, for thick canopies (i.e. Λ ≤ 1, black points in Fig. 6(left)). The data regression of *f*/*λ* measured under shallow submergence (Λ ≤ 1) results in the following expression

13 $$\frac{f}{\lambda }\sim {(\frac{1}{R{e}^{\star }})}^{\beta },\quad \beta ={\mathscr{O}}(1)$$

with

14 $$R{e}^{\star }=\frac{\rho {\hat{U}}_{b}(H+L)}{{\mu }_{t}}.$$

The proposed scaling (13) suggests that the friction factor appropriately normalized by the dimensionless permeability follows a universal scaling for thick canopies (Λ ≤ 1, or canopies with small submergence). The deviation of Cassan data (grey points in Fig. 6(left)) is expected since they fall in the category of thin porous media (${\rm{\Lambda }}\gg 1$) or canopy with high submergence, where boundary layer-type velocity profiles develop. The fitted exponent *β* ≈ 0.86 corresponds to a less than quadratic relationship between the drag force and the mean flow, specifically $D\sim {\hat{U}}_{b}^{1.86}$. This is consistent with the scaling experimentally measured by Sand-Jensen^40^, who found exponents in the range (1.3, 1.9) and Armanini *et al*.^19^, who found an almost linear relationship between the drag force and the velocity for flexible submerged canopies.

Since $\lambda ={\mathscr{O}}\mathrm{(1)}$ for the data analyzed, we hypothesize that $\lambda ={\mathscr{O}}\mathrm{(1)}$ for most submerged canopies. Under this hypothesis, (13) can be further simplified to

15 $$f\sim {(\frac{1}{R{e}^{\star }})}^{\gamma },\quad \gamma ={\mathscr{O}}(1).$$

In Fig. 6(right) we plot the experimentally measured friction factor *f* in terms of $R{e}^{\star }$ for a much wider dataset including rigid and flexible vegetation. Specifically, Fig. 6(right) includes the dataset of Table 1 in addition to 132 data points analyzed by Poggi, *et al*. in their Table 1^30^ and 13 profiles measured across different sections of the Babolrod River, Western Daronkola in Mazandaran, Iran^70^ for a total of 164 points. A description of the additional data is included in the Methods Section. In absence of estimates of the parameter Λ, we classify the data in terms of the canopy submergence (*H* + *L*)/*H*, where the dark grey points in Fig. 6(right) have submergence greater than 10. The data analysis suggests that the scaling (15) provides a good estimate of the order of magnitude of Darcy friction factor for both rigid and flexible vegetation across different canopy morphologies for submergence lower than 5.

## Conclusions

Accurate estimates of the impact of vegetation on flow resistance in riverine systems are critical to assess river management and restoration and to ensure aquatic habitat resilience and health. A common way to evaluate the quality of aquatic habitats is to determine whether selected hydraulic parameters (such as the water depth and flow velocity) fall within a range considered suitable for the biological needs of a specific organism. Since submerged vegetation reduces the mean velocity in the channel, accurate predictions of vegetation-mediated flow resistance is crucial to monitor the biotic diversity, to preserve a healthy life for the macro-invertebrates and fishes that find shelter in the submerged canopies, and to control the deposition of inorganic sediments in marshes^71^. Characterization of flow resistance for channels with topologically complex vegetation has proven elusive, though. On one hand, attempts to correlate the drag coefficient to relevant flow and vegetation parameters has opened a Pandora’s box in which the intertwining of topological and dynamical controls on friction has been hard to disentangle. Similarly, simple experimental formulas such as Manning’s, have proven to have poor predictive capability, especially in channels with complex morphology^34^. On the other hand, a few studies have demonstrated unique universal features of flows over obstructions of disparate types and topologies^48,68^ ranging from submerged aquatic vegetation canopies, terrestrial vegetation canopies, coral reefs, and dense porous media, suggesting that reduced-complexity models may be successful in capturing some of the universal features of such flows^48^.

Differently from other approaches where the additional resistance introduced by canopies is modeled through a quadratic friction parametrization, we model the flow resistance generated by the canopy as a Darcy-type resistance, corrected to account for vortices penetration in the superior portion of the canopy layer through an estimate of the effective turbulent viscosity, *μ*_*t*_^11^. We first demonstrate that the analytical expressions of Battiato and Rubol^11^ well predict the velocity profiles, volumetric flow rates and the flow resistance measured across a wide range of natural meadows with very different morphologies. The comparison focuses on profiles that do not present a secondary maximum of velocity in the lower part of the canopies, even though generalizations to this scenario are relatively straightforward and focus of current investigations. The model predictions compare well with the global resistance to flow under different conditions of plant density and morphologies.

Finally, we propose and test a novel universal scaling for the Darcian friction factor *f*. We demonstrate that *f* for canopies with low submergence is controlled primarily by the canopy permeability (1/*λ*) and a modified Reynolds number $R{e}^{\star }$. Importantly, we show that *f*/*λ* scales universally with the inverse of $R{e}^{\star }$, i.e. the ratio *f*/*λ* is independent of the canopy density and shape. Additionally, since for most canopies analyzed $\lambda ={\mathscr{O}}\mathrm{(1)}$, we show that $f\sim 1/R{e}^{\star }$ still exhibit a universal behavior. This result has important implications in that it allows one to quite accurately estimate the magnitude of the friction factor through measurements of discharge and slope and by means of remote sensing and acoustic techniques (to determine water level and canopy submergence). This result could pave the way to creating large scale maps of riverine vegetation-mediated friction factor estimates.

The main conclusions of this study can be summarized as follows:

- Modeling submerged vegetation as a porous medium allows one to describe the flow velocity, discharge and friction factors in channels with canopy with complex morphology including grasses, woody vegetation bushes as well as prone vegetation.
- Closed-form expressions derived for non-deformable porous media can be successfully adopted to describe turbulent flow over flexible canopies when (i) deflected canopy height data are available and (ii) the velocity profile does not present a secondary maximum of velocity in the lower part of the canopies due to nonuniform plant mass distribution.
- For canopies with low submergence, the product between the Darcy friction factor and the canopy layer dimensionless permeability scales with the inverse of the bulk Reynolds number rescaled by the fluid to the turbulent viscosity ratio. The scaling-law solely depends on canopy height, water level and channel geometry, while it is universal across vegetation morphology. This provides a valuable tool to assess habitats sustainability associated to hydro-dynamical conditions.

## Methods

### Dataset used for Data Validation

The velocity profile and discharge dataset from Table 1 were collected from a variety of flume experiments with the exception of Cassan’s data^56^ (Cassan11, Cassan12, Cassan21, Cassan22) which investigated the flow in a real vegetated channel. The experiments included plants with morphological shapes that resembled both riverbed and riparian vegetation. Plants included grasses (e.g., eelgrass, cordgrass and barley), woody vegetation (e.g., poplars) and bushes (e.g., willows) as well as macrophytes and briophytes. The sketch of the plants morphology is included in Fig. 1, while a brief description of the plant shape is included in the following paragraph ‘Dataset and Plant Morphologies’. The input parameter for the simulation - (deflected) canopy height, water level and channel slope - are listed in Table 2. Given the uncertainty in the reduced von Kármán constant for vegetated flows, data were compared with the model of Battiato and Rubol^11^ using both one-fitting and two-fitting parameters. In the former we fit the canopy permeability only (black lines in Figs 2 and 3) while the von Kármán constant is set to *κ* = 0.19 for all the model predictions; the latter considers both the permeability and the von Kármán constant as fitting parameters (gray lines in the Figs 2 and 3). Only for submergence greater than five (i.e., datasets CassanS11, CassanS12, CassanS21, CassanS22, and Shi20), the von Kármán constant was set to *κ* = 0.41 in the one-parameter model. The two-fitting parameter model was then used to predict the velocity profiles of Fig. 4, while the one-fitting parameter model was used to determine the flow rate values illustrated in Fig. 5. The values of the fitting parameters and the main outputs are included in Table 2.

To evaluate the error between the model predictions and the experimental data, we calculate the mean relative error for both the one-fitting and the two-fitting parameter model using the following equation:

16 $$Error=\frac{1}{N}\sum _{i=1}^{N}|\frac{{\hat{u}}_{i,j}-{\hat{u}}_{i,{\rm{D}}{\rm{a}}{\rm{t}}{\rm{a}}}}{{\hat{u}}_{i,{\rm{D}}{\rm{a}}{\rm{t}}{\rm{a}}}}|,\quad j=\{1p,\,2p\}$$

where *N* is the total number of data points and ${\hat{u}}_{i}$ is the velocity. The datasets Shi20, Shi40 and Shi60^60^ presented multiple velocity measurements at the same location: we used the averaged profile obtained by averaging the velocity values of the points at the same vertical coordinate. For Fig. 6(right) we considered canopies consisting of arrays of dowels, plastic or real vegetation. More precisely, in addition to the 24 points of Table 1, we considered 132 points from the Table 1 by Poggi *et al*.^30^ (55 from rigid and 77 from flexible canopies), plus 13 profiles measured across different sections of the Babolrod River, Western Daronkola in Mazandaran, Iran^70^. The vegetation measured in the work of Miyab *et al*.^70^ had an average height of 19 cm and an average density of 331 stems per meter long. The total number of points in Fig. 6(right) is 164 (due to a 5 point overlap between Table 1 of the present study and Table 1 by Poggi *et al*.^30^).

### Dataset and Plant Morphologies

#### Plastic vegetation

Nepf *et al*.^55^ use a plastic plant prototype resembling eelgrass (e.g. *Zostera marina*) made of 0.025-cm thick vinyl plastic with six 0.3 cm wide blades. Wilson *et al*.^63^ use a plastic prototype of a marine kelp *Laminaria hyperboerea* which also resembles aquatic macrophytes. The prototype consists of a stipe with a basal diameter of 4 mm and 85 mm height with a frond 70 mm height and 100 mm wide. Baptist *et al*.^62^ use plastic aquarium plants, AQUASCAPERS, from Metaframe Corporation, USA, type Anacharis (Egeria densa) X-large that resembled *Elgeria Densa* large-flowered waterweed. Siniscalchi *et al*.^58^ investigate the flow over a finite-size vegetation patch. The patch consists of 53 artificial flexible plants, with a spacing of 20 cm between them. Plants resemble young poplars growing on floodplains and are composed of a 3 mm thick coated wire stem, a blossom, and four branches with three leaves each.

#### Real vegetation

Righetti *et al*.^57^ investigate the flow over real bushes, willows of 70 cm height (*Salix pentandra*) that are present on banks, floodplains and also in riverbeds. Shucksmith *et al*.^61^ investigate the flow over growing Carex, an evergreen perennial dense leafy plant with solid stems and flat leaves, commonly found in European rivers. Velasco *et al*.^59^ consider high density canopy (22500 stem/m^2^) made with natural grass (cultivated barley). The experiments by Shi *et al*.^60^ are run using *Spartina anglica*, or cordgrass, collected from Humber Estuary in the United Kingdom. The plants were cut to an average height of 300 mm and had an average diameter or 4 mm. Plants were further cut to different percentage of this height in the simulation (see the values in Table 2). The irrigation channel of Cassan *et al*.^56^ is colonized by briophytes (*Fortinalis antypiretica* with stems up to few centimeters long and small leaves (1–4 mm) and long macrophytes (*Ranunculus fuitanis*) that were bent by the flow occupying a layer of 5–10 cm above the bed. Data are collected at two different gage stations with Station 1 mainly colonized by macrophytes and Station 2 presenting a more uniform vegetation distribution. Measurements at both stations were collected in both spring and summer. The main input and output are summarized in Table 2.

#### Dataset of Fig. 6(right)

The right panel of Fig. 6 contains 164 points including flexible and rigid vegetation. We refer to the Appendix B of Poggi *et al*.^30^ for a detailed description of the mophology used in their work.

#### Data Availability

The datasets generated during, and/or analyzed in, the current study are available from the corresponding author on reasonable request.

### Buckingham Π Theorem and Universal Scaling

A standard approach to find self-similar solutions to boundary value problems is to use dimensional analysis. The drag force *D* can be written as a function of both geometric (*K*, *L*, *H*), and dynamic (${\hat{U}}_{b}$, *μ*_*t*_, *μ* and *ρ*) parameters, i.e.

17 $$D={{\varphi }}_{1}({\hat{U}}_{b},K,{\mu }_{t},\mu ,L,H,\rho )$$

where ${\hat{U}}_{b}$ is the mean (bulk) velocity, *K* is the canopy permeability, *ρ*, *μ* and *μ*_*t*_ are the density, viscosity and turbulent viscosity of the flow. Since the flow is turbulent, it is expected that the dynamics is mainly controlled by *ρ* and *μ*_*t*_. Therefore,

18 $$D={{\varphi }}_{2}({\hat{U}}_{b},K,{\mu }_{t},L,H,\rho \mathrm{)}.$$

Using Buckingham Π’s theorem and selecting *μ*_*t*_, ${\hat{U}}_{b}$ and ${\mathcal L}$ as repeating variables, where ${\mathcal L} =H+L$ is a characteristic length scale of the system, we obtain

19 $${{\rm{\Pi }}}_{D}={\varphi }_{3}(\frac{K}{{{\mathscr{L}}}^{2}},\frac{L}{{\mathscr{L}}},R{e}^{\star }),$$

or, equivalently,

20 $${{\rm{\Pi }}}_{D}={\varphi }_{4}(\frac{K}{{{\mathscr{L}}}^{2}},\frac{H}{{\mathscr{L}}},R{e}^{\star }).$$

In (19) and (20),

21 $${{\rm{\Pi }}}_{D}=\frac{D}{{\mu }_{t}{\hat{U}}_{b}{\mathscr{L}}}\quad \,{\rm{a}}{\rm{n}}{\rm{d}}\quad R{e}^{\star }=\frac{\rho {\hat{U}}_{b}{\mathscr{L}}}{{\mu }_{t}}$$

are the dimensionless drag and a modified Reynolds number, defined as the product between $Re=\rho {\hat{U}}_{b} {\mathcal L} /\mu$ and the fluid to turbulent viscosity ratio *η* = *μ*/*μ*_*t*_, respectively. Recombination of the dimensionless parameters in (20) leads to

22 $${{\rm{\Pi }}}_{D}={\varphi }_{4}(\lambda ,\delta ,R{e}^{\star }),$$

with

23 $$\lambda \,:=\frac{H}{\sqrt{K}}\quad {\rm{and}}\quad \delta \,:=\frac{L}{H}.$$

The dimensionless parameter ${\rm{\Lambda }}:=\delta \lambda =L/\sqrt{K}$ provides a dynamic classification between thin (${\rm{\Lambda }}\gg 1$), or high submergence, and thick (Λ ≤ 1), or low submergence, canopy layers^50,69^. The former exhibit boundary layer features while the latter present a characteristic inflection point in their mean velocity profiles. Importantly, it has been shown that in thick (Λ ≤ 1) and thin (${\rm{\Lambda }}\gg 1$) porous layers flow and transport are controlled either by the dimensionless height of the obstruction, *δ* (or its submergence) or by the obstruction dimensionless permeability *λ*^−1^, respectively^50,69^, i.e.

24 $${{\rm{\Pi }}}_{D}=\psi (\delta ,R{e}^{\star })\quad {\rm{f}}{\rm{o}}{\rm{r}}\quad {\rm{\Lambda }}\gg 1,$$

and

25 $${{\rm{\Pi }}}_{D}=\varphi (\lambda ,R{e}^{\star })\quad {\rm{f}}{\rm{o}}{\rm{r}}\quad {\rm{\Lambda }}\le 1.$$

Since the data set considered in Figs 2 and 3 is predominantly characterized by thick porous media, i.e. small submergence (except for Cassan’s profiles), we employ (25) to identify a possible scaling behaviour. We postulate the existence of self-similarity of the second kind in the form

26 $${{\rm{\Pi }}}_{D}={\lambda }^{\alpha }\varphi (R{e}^{\star }).$$

If ${{\rm{l}}{\rm{i}}{\rm{m}}}_{R{e}^{\ast }\to 0}\varphi (R{e}^{\star })={\rm{F}}{\rm{i}}{\rm{n}}{\rm{i}}{\rm{t}}{\rm{e}}\ne 0$, then the drag force *D* scales as $D\sim {\lambda }^{\alpha }{\mu }_{t}{\hat{U}}_{b} {\mathcal L}$ as $R{e}^{\star }\to 0$, and the drag coefficient

27 $${C}_{D}\,:=\frac{2D}{\rho {\hat{U}}_{b}^{2}{ {\mathcal L} }^{2}}$$

as

28 $${C}_{D}\sim \frac{{\lambda }^{\alpha }}{R{e}^{\star }}.$$

To test the proposed scaling (28) under the hypothesis that most of the resistance offered by vegetation is due to friction, i.e. ${C}_{D}\sim f$, we plot *f*/*λ* as a function of $R{e}^{\star }$ where *f* is the friction factor and *α* = 1, see Fig. 6(left). The data analysis, discussed in the Results and Discussion Section, confirms that *f*/*λ* scales with the inverse of $R{e}^{\star }$, the modified Reynolds number for thick canopies (i.e. Λ ≤ 1, black points in Fig. 6(left)). The data regression of *f*/*λ* measured under shallow submergence (Λ ≤ 1) results in the following universal expression

29 $$\frac{f}{\lambda }\sim {(\frac{1}{R{e}^{\star }})}^{\beta },\quad \beta ={\mathscr{O}}(1)$$

with

30 $$Re=\frac{\rho {\hat{U}}_{b}(H+L)}{{\mu }_{t}}.$$

where $${\mu }_{t}=\rho \kappa H{\hat{u}}_{\tau }$$ and $${\hat{u}}_{\tau }=\sqrt{g{S}_{0}L}$$.
